# Comparison between volume and surface coils for pig cardiac metabolism studies with hyperpolarized 13C MRS

**DOI:** 10.1186/1532-429X-13-S1-P343

**Published:** 2011-02-02

**Authors:** Giulio Giovannetti, Maria Filomena Santarelli, Francesca Frijia, Luca Menichetti, Jan Henrik Ardenkjaer-Larsen, Daniele De Marchi, Valentina Hartwig, Vincenzo Positano, Luigi Landini, Massimo Lombardi

**Affiliations:** 1 CNR-Institute of Clinical Physiology, Pisa, Italy; 2 Fondazione “G. Monasterio” CNR-Regione Toscana, Pisa, Italy; 3 GE Healthcare, 3400 Hillerod, Denmark; 4 Department of Information Engineering, University of Pisa, Pisa, Italy

## Introduction

Cardiac metabolism assessment with hyperpolarized 13C in pig models requires dedicated coils design capable to provide large sensitivity regions.

This work presents a comparison between a quadrature birdcage coil and a circular coil both designed for hyperpolarized studies of the heart in large animal models with a clinical 3T scanner. The results are presented as Signal-to-Noise Ratio (SNR)-vs-depth profiles using experimental SNR extracted from the (1-13C) acetate phantom chemical shift image (CSI).

## Methods

Birdcage are widely used as transmitter and receiver coils, since its ability to produce circular polarized field that increases SNR by a factor of with respect to linear polarization while providing a highly homogenous field. Surface coils are usually much smaller than the volume coils and have higher SNR because they receive noises only from nearby regions. However, they are mainly used as receive coils since their relatively poor field homogeneity.

In this work we compared a quadrature birdcage coil (Rapid Biomedical, Würzburg, Germany) and a home-made transmit-receive circular coil, whose design was performed taking into account for the sample dimension and shape (pig heart).

The coils testing was performed using a 10 g (1-13C) acetate cylindrical phantom whose dimensions roughly matches the pig heart. The experiments were performed with a 3T GE Signa HDx (GE Healthcare, Waukesha, WI, USA) clinical scanner. CSI of the (1-13C) acetate phantom were acquired in order to evaluate the SNR profiles vs depth. The CSI data were acquired using an elliptic FIDCSI sequence with the following parameters: FOV 120 mm, 16x16 matrix, slice thickness 2 cm, FA 12°, TR 80 ms, and bandwidth 5000 Hz.

## Results and discussion

**Figure 1:**
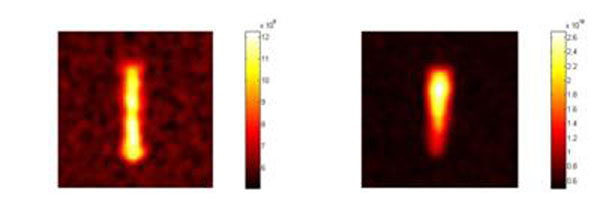


**Figure 2:**
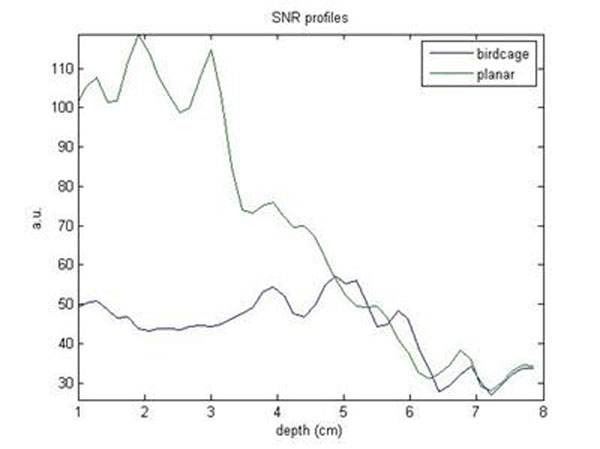


Figure 1 shows the SNR trends along the (1-13C) acetate test phantom for the two coils. Although the rapid magnetic field falling off, the circular coil provides higher SNR along a wide phantom length, while at the maximum depth of desired imaging the two SNR values are perfectly comparable. Figure 2 shows an example of CSI data acquired with the birdcage (on the left) and the surface coil (on the right).

## Conclusion

We have demonstrated that the use of a home-built surface coil, theoretically designed in order to achieve a good penetration in deep sample regions, can provide good SNR with respect to the one guaranteed by a quadrature birdcage coil. The comparison of SNR-vs-depth profiles for the two coils are valid even for hyperpolarized 13C studies.

